# Vancomycin Exposure Dynamics and Clinical Outcomes in Critically Ill Patients: A Retrospective Cohort Study

**DOI:** 10.3390/antibiotics15060573

**Published:** 2026-06-04

**Authors:** Mohamad Amer Nashtar, Jutta Dedy, Stamatina Georgitsi, Gizem Garipoglu, Asterios Tzalavras, Ali Canbay, Tim Rahmel, Despoina Koulenti, Claire Roger, Antonios Katsounas

**Affiliations:** 1 Ruhr University Bochum, Knappschaft Kliniken University Hospital Bochum, Department of Medicine, 44892 Bochum, Germany; mohamad.nashtar@knappschaft-kliniken.de (M.A.N.);; 2 Department of Pharmacy, University Hospital Essen, University of Duisburg Essen, 45147 Essen, Germany; 3 Ruhr University Bochum, Knappschaft Kliniken University Hospital Bochum, Department of Anesthesiology, Intensive Care and Pain Therapy, 44892 Bochum, Germany; 4 Department of Critical Care Medicine, King’s College Hospital NHS Foundation Trust, London SE5 9RS, UK; 5 UR-UM103 IMAGINE, Division of Anesthesia Critical Care, Pain and Emergency Medicine, Nîmes University Hospital, University of Montpellier, 34090 Montpellier, France; claire.roger@chu-nimes.fr

**Keywords:** vancomycin, therapeutic drug monitoring (TDM), time in therapeutic range (TIR), volatility index, augmented renal clearance (ARC), acute kidney injury (AKI)

## Abstract

**Objectives:** Vancomycin is crucial for treating severe Gram-positive infections, but its narrow therapeutic index complicates dosing. Trough monitoring may inadequately reflect exposure, while AUC-guided dosing, although recommended, is often impractical. Alternative metrics such as the time in therapeutic range (TIR) and volatility index may reflect exposure dynamics. Augmented renal clearance (ARC) further challenges vancomycin therapy in Intensive Care Unit (ICU) settings. This study evaluated trough-based exposure metrics and their associations with ICU mortality and acute kidney injury (AKI). **Methods:** We retrospectively analyzed 109 ICU patients with sepsis receiving vancomycin. Exposure was assessed using mean trough concentrations, TIR (proportion of troughs within predefined ranges), and the volatility index, defined as the intra-individual standard deviation divided by the mean trough concentration (SD/mean). Outcomes were ICU mortality and AKI. Associations were evaluated using multivariable regression, bootstrap resampling, and restricted cubic splines. **Results:** TIR _>15_ was independently associated with higher mortality (adjusted OR 3.88; *p* = 0.0326) and AKI stage II–III (adjusted OR 5.63; *p* = 0.0068). Higher mean troughs correlated with AKI stage II–III, whereas higher volatility showed an inverse association (adjusted OR 0.15; *p* = 0.0240). ARC (4.6%) occurred exclusively in younger patients and predicted subtherapeutic exposure (TIR _<10_, *p* = 0.0485). **Conclusions:** Sustained troughs >15 mg/L were associated with mortality and nephrotoxicity, while the most favorable outcomes were descriptively observed at mean trough levels of approximately 8–12 mg/L, suggesting a possible narrow exposure range that requires prospective validation. These findings highlight the limitations of trough-based monitoring alone; the trough-derived metrics should be regarded as exploratory rather than validated decision-making tools.

## 1. Introduction

Vancomycin remains a cornerstone in the treatment of severe Gram-positive infections, particularly methicillin-resistant *Staphylococcus aureus* (MRSA), in critically ill patients. Due to its narrow therapeutic index and nephrotoxic potential, therapeutic drug monitoring (TDM) is essential to balance efficacy and safety [1]. Traditionally, vancomycin dosing has been guided by trough concentrations, which remain widely used; however, accumulating evidence has questioned their reliability as surrogates for overall exposure and toxicity risk [2]. In 2020, the ASHP–IDSA–PIDS–SIDP consensus guideline recommended a shift toward Area Under the Concentration–Time Curve/Minimum Inhibitory Concentration (AUC/MIC)-guided dosing as the new standard of care, targeting an AUC of 400–600 mg·h/L (assuming MIC = 1 mg/L) to optimize outcomes while reducing nephrotoxicity [2]. However, direct AUC estimation requires specialized tools and is not always feasible in routine practice. Because full Bayesian AUC estimation is not consistently available in all clinical settings, serial trough measurements may still provide useful longitudinal information beyond isolated trough values. In this context, trough-derived metrics such as time in therapeutic range (TIR) and the volatility index can serve as pragmatic surrogate descriptors of exposure dynamics rather than replacements for AUC/MIC-guided monitoring [3,4,5]. TIR summarizes the relative frequency of trough concentrations within or above predefined ranges, thereby helping to distinguish isolated excursions from recurrent or sustained exposure patterns. The volatility index, calculated as the intra-individual standard deviation divided by the mean concentration (SD/mean), captures relative within-patient variability and may reflect dynamic changes in renal clearance or dose adaptation. Together, these metrics may provide complementary exploratory information on longitudinal vancomycin exposure when repeated trough measurements are available [6].

Another critical determinant of vancomycin pharmacokinetics in intensive care unit (ICU) settings is augmented renal clearance (ARC), typically defined as creatinine clearance >130 mL/min/1.73 m^2^ [7]. ARC refers to enhanced renal elimination of solutes beyond normal physiological levels and is increasingly recognized as a frequent phenomenon in critically ill patients, particularly in younger individuals, trauma cases, and those with sepsis [8,9]. ARC represents a key pathophysiological alteration in intensive care, as it can markedly increase the clearance of renally eliminated drugs. Consequently, ARC has major implications for antibiotic pharmacokinetics, often leading to subtherapeutic plasma concentrations and treatment failure when standard dosing regimens are applied [10,11,12,13]. Conversely, impaired renal function predisposes to drug accumulation and nephrotoxicity. However, vancomycin exposure is more broadly determined by overall renal function across the full spectrum of glomerular filtration, with ARC representing an extreme phenotype rather than the predominant determinant. Therefore, understanding the interplay between vancomycin exposure metrics, ARC, and clinical outcomes is essential for refining individualized dosing strategies in this high-risk population.

Against this background, we conducted a retrospective study in critically ill patients with sepsis to evaluate vancomycin exposure based on mean vancomycin trough concentrations (VTCs) and two trough-derived surrogate measures (TIR and volatility index). We investigated their association with ICU mortality and acute kidney injury (AKI) and explored the potential impact of ARC on vancomycin pharmacokinetics. By evaluating these trough-derived exposure measures in relation to patient outcomes, this study aims to provide clinically relevant insights into vancomycin exposure patterns in critically ill patients where full AUC-guided dosing may not be feasible.

## 2. Results

### 2.1. Patient Characteristics

Of the 109 patients included, 51 (46.8%) were admitted via the Department of Gastroenterology and Hepatology, while 58 (53.2%) were admitted via the Department of Nephrology. The mean age of the study population was 60 years. The majority of patients were male (n = 76; 69.7%). The median duration of vancomycin therapy was 6 days. All patients were admitted with sepsis, most frequently due to pneumonia (n = 56, 51.4%), followed by urosepsis (n = 26, 23.9%), sepsis of undetermined focus (n = 12, 11.0%), and other infectious causes (n = 15, 13.8%). All patients received vancomycin therapy. The mean vancomycin trough concentration (VTC) was 13.1 ± 4.7 mg/L, and the volatility index was 0.31 ± 0.27. We additionally assessed the TIR for different exposure windows, with mean values of 0.51 ± 0.35 for TIR _10–20_, 0.22 ± 0.26 for TIR _15–20_, and 0.30 ± 0.31 for TIR _15–25_. Overall ICU mortality during the index ICU stay was 47.7% (n = 52). ARC was observed in 5 patients (4.6%). During the course of therapy, AKI occurred frequently, with 11 patients (10.1%) meeting KDIGO criteria for AKI stage I and 48 patients (44.0%) fulfilling criteria for AKI stage II–III. Older patients showed significantly lower eGFR values compared to younger tertiles, while ARC was observed exclusively in the youngest group (≤54 years, *p* = 0.0061). This age distribution is consistent with the known predominance of ARC in younger critically ill patients; however, given the small number of ARC cases (n = 5), this finding should be interpreted descriptively. Detection of vancomycin-susceptible bacteria decreased with age, being most frequent in younger and middle tertiles (62.2% each) but only 37.1% in the oldest group (*p* = 0.0493). No significant differences were found across age groups regarding mortality, AKI, treatment duration, or vancomycin exposure parameters (mean trough, TIR, volatility index). A detailed overview of baseline characteristics is provided in Table 1. The distribution of isolated bacteria and their respective sources are shown in Supplementary Tables S1 and S2, summarizing vancomycin-susceptible isolates and Gram-negative isolates, respectively.

### 2.2. Impact of Vancomycin Exposure on Clinical Outcomes

TIR _>15_ was associated with increased ICU mortality. In univariate analysis, TIR _>15_ was linked to higher mortality (OR 3.12; 95% CI 1.15–10.85; *p* = 0.0417). This association remained significant after adjustment for sex and age (OR 3.88; 95% CI 1.12–14.44; *p* = 0.0326).

**Figure 1:**
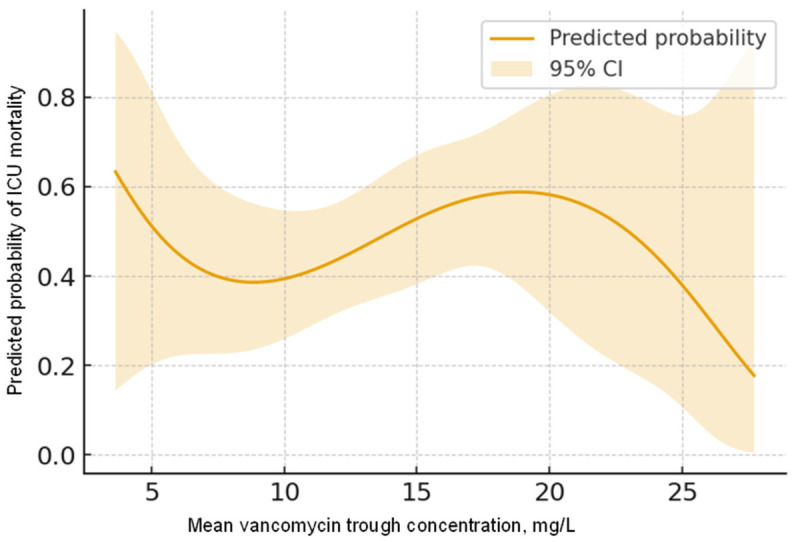
Restricted cubic spline: Mean vancomycin trough concentration vs. ICU mortality. Restricted cubic spline model illustrating the association between mean vancomycin trough concentrations and ICU mortality. The solid line shows the predicted probability of ICU mortality, and the shaded area represents the 95% confidence interval. A total of 52 patients (47.7%) died during vancomycin therapy. The lowest mortality was observed in patients with intermediate vancomycin exposure, particularly in those with mean trough concentrations between 7.6–11.6 mg/L (empirical bin analysis, ~33–35%) and 9.8–12.6 mg/L (quartile analysis, ~42%).

**Figure 2:**
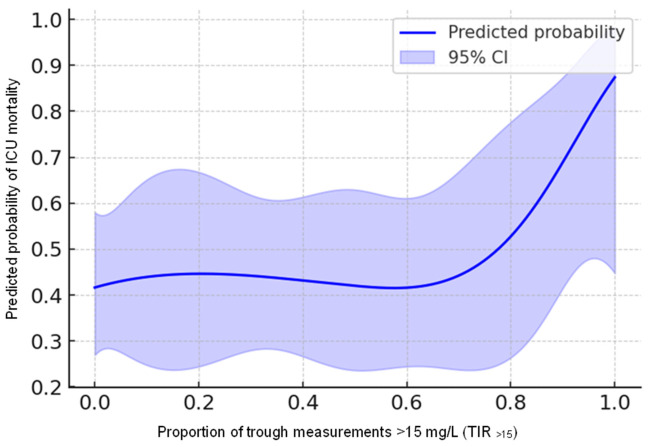
Restricted cubic spline: TIR _>15_ vs. ICU mortality. Restricted cubic spline model depicting the association between time in therapeutic range (TIR) _>15_ and ICU mortality. TIR _>15_ was defined as the proportion (0–1) of available trough measurements exceeding 15 mg/L and does not represent a time-weighted exposure. The solid line represents the predicted probability of ICU mortality, and the shaded area indicates the 95% confidence interval. A total of 52 patients (47.7%) died during vancomycin therapy. Sustained vancomycin exposure above 15 mg/L was associated with ICU mortality (adjusted OR 3.88, 95% CI 1.12–14.44, *p* = 0.0326).

Descriptive analyses suggested a U-shaped relationship, with the lowest mortality observed in patients with intermediate vancomycin exposure, particularly in those with mean trough concentrations between 7.6–11.6 mg/L (empirical bin analysis, ~33–35%) and 9.8–12.6 mg/L (quartile analysis, ~42%) (Figure 1 and Figure 2). To assess the robustness of the association between TIR _>15_ and mortality, we performed a bootstrap resampling analysis with 1000 iterations. In the univariable logistic regression, TIR _>15_ remained significantly associated with increased mortality (bootstrap mean OR 4.37, 95% CI 1.11–13.23, *p* = 0.0240). After adjustment for age and sex, this association persisted with similar magnitude and significance (bootstrap mean OR 5.26, 95% CI 1.23–15.18, *p* = 0.0200). These findings confirm the stability of the observed effect across repeated resampling and support the robustness of the primary analysis.

**Figure 3:**
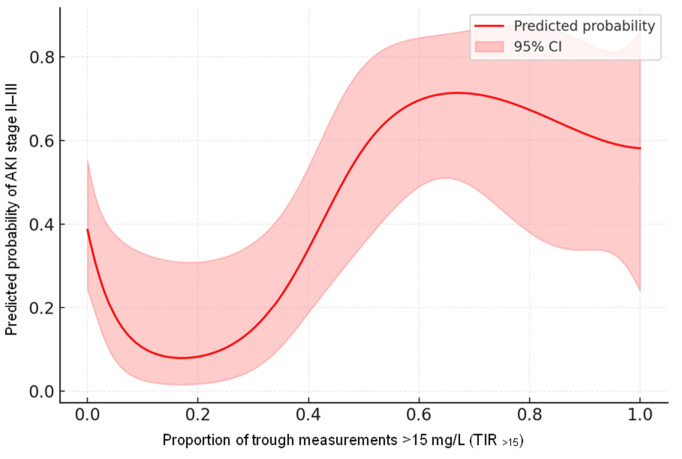
Restricted cubic spline: TIR _>15_ vs. AKI stage II–III. Restricted cubic spline model depicting the association between time in therapeutic range (TIR) _>15_ and AKI stage II–III. TIR _>15_ was defined as the proportion (0–1) of available trough measurements exceeding 15 mg/L and does not represent a time-weighted exposure. The solid line represents the predicted probability of AKI stage II–III, and the shaded area indicates the 95% confidence interval. A total of 48 patients (44.0%) developed AKI stage II–III during vancomycin therapy. Sustained vancomycin exposure above 15 mg/L was associated with AKI stage II–III (adjusted OR 5.63, 95% CI 1.60–21.52, *p* = 0.0068).

**Figure 4:**
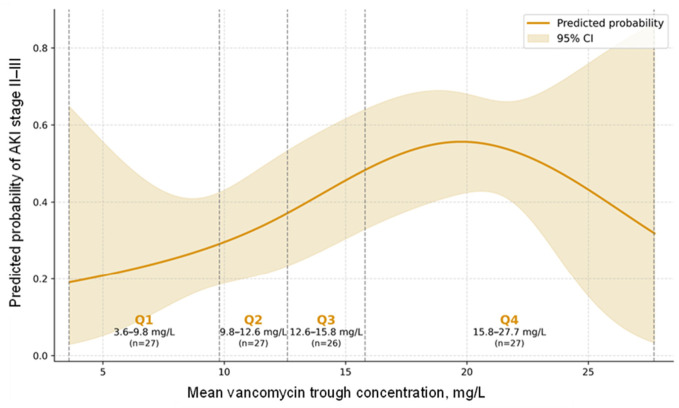
Restricted cubic spline: Mean vancomycin trough concentration vs. AKI stage II–III. Restricted cubic spline illustrating the association between mean vancomycin trough concentrations and the occurrence of AKI stage II–III. The solid orange line shows the predicted probability of AKI stage II–III, and the shaded area represents the 95% confidence interval. Quartile boundaries of mean vancomycin trough concentrations are indicated directly on the *x*-axis for clarity. A total of 48 patients (44.0%) developed AKI stage II–III during vancomycin therapy. Higher vancomycin trough concentrations (VTCs) were significantly associated with AKI stage II–III (adjusted OR 1.122, 95% CI 1.026–1.238, *p* = 0.0114). The lowest incidence of AKI stage II–III was observed in the first quartile (3.6–9.8 mg/L; 25.9%), whereas the highest incidence occurred in the fourth quartile (15.8–27.7 mg/L; 59.3%).

TIR _>15_ was significantly associated with the occurrence of AKI. In univariate logistic regression, TIR _>15_ was associated with AKI stage II–III (OR 5.38; 95% CI 1.58–19.71; *p* = 0.0067). This association remained robust in the bootstrap resampling analysis with 1000 iterations, yielding a mean OR of 9.17 (95% CI 1.74–35.99, *p* < 0.001). After adjustment for age and sex, the association between TIR _>15_ and AKI stage II–III persisted (OR 5.63; 95% CI 1.60–21.52; *p* = 0.0068). The bootstrap sensitivity analysis confirmed this finding, with a mean OR of 9.13 (95% CI 1.56–37.76, *p* = 0.0060) (Figure 3). In univariate analysis, higher mean VTCs were significantly associated with AKI stage II–III (OR 1.123, 95% CI 1.029–1.236, *p* = 0.0084). After adjustment for sex and age, the association remained significant (OR 1.122, 95% CI 1.026–1.238, *p* = 0.0114). In quartile analysis, the lowest incidence of AKI stage II–III was observed in the first quartile (3.6–9.8 mg/L; 25.9%), whereas the highest incidence occurred in the fourth quartile (15.8–27.7 mg/L; 59.3%) (Figure 4).

Restricted cubic spline analyses did not reveal significant non-linear associations between mean VTCs or TIR _>15_ and ICU mortality or AKI stage II–III (likelihood ratio test *p* > 0.05 for each). Given the modest sample size, wide confidence intervals, and limited number of patients at the extremes of vancomycin exposure, the statistical power to detect non-linearity was limited. Accordingly, the apparent U-shaped exposure–mortality pattern should be interpreted as descriptive and exploratory rather than as a statistically confirmed non-linear relationship.

The volatility index was inversely associated with the occurrence of AKI. In univariate logistic regression, a higher volatility index was associated with a reduced risk of AKI stage II–III (OR 0.13; 95% CI 0.02–0.68; *p* = 0.0140). This association remained significant after adjustment for sex and age (OR 0.15; 95% CI 0.02–0.79; *p* = 0.0240). In addition, the volatility index was significantly associated with ARC (OR 11.49; 95% CI 2.77–150.2; *p* = 0.0442). However, this association was attenuated and no longer statistically significant after adjustment for age.

Age was significantly associated with vancomycin exposure–related parameters. Higher age was linked to a lower volatility index (β −0.0050; 95% CI −0.0088 to −0.0013; *p* = 0.0084) and a lower probability of TIR _<10_ (β −0.0058; 95% CI −0.0107 to −0.0009; *p* = 0.0200). In addition, age was negatively associated with ARC (OR 0.855; 95% CI 0.760–0.927; *p* < 0.0001).

Increased eGFR was positively associated with TIR _<10_ (β 0.0026; 95% CI 0.0009–0.0043; *p* = 0.0030) and with the volatility index (β 0.0016; 95% CI 0.0003–0.0029; *p* = 0.0160). As expected, ARC was significantly associated with subtherapeutic vancomycin exposure, defined as TIR _<10_ (OR 13.28; 95% CI 1.02–312.6; *p* = 0.0485).

Detection of vancomycin-resistant *Enterococcus* (VRE) was associated with renal outcomes. Current detection was significantly associated with AKI stage II–III (OR 8.57; 95% CI 1.39–165.0; *p* = 0.0179), as was a history of VRE detection (OR 3.65; 95% CI 1.13–14.11; *p* = 0.0302). After adjustment for sex and age, both associations remained significant, with current VRE detection (OR 9.13; 95% CI 1.46–177.4; *p* = 0.0157) and any VRE history (OR 3.82; 95% CI 1.15–15.15; *p* = 0.0276) showing independent associations with AKI stage II–III.

No significant associations were observed between ICU mortality and age, sex, treatment duration, volatility index, AKI stage I, AKI stage II–III, ARC, VRE status, or detection of Gram-negative bacteria (Supplementary Table S3).

## 3. Discussion

In this retrospective study of 109 critically ill septic patients, descriptive analyses suggested a possible U-shaped relationship between mean VTCs and ICU mortality, with the lowest mortality observed at mean trough levels around 8–12 mg/L and higher mortality observed below and above this range. Importantly, TIR _>15_ was consistently associated with higher mortality and an increased risk of AKI stage II–III. These findings suggest that cumulative exposure above 15 mg/L may reflect sustained overexposure rather than isolated peaks. Clinically, prolonged or frequent supratherapeutic levels—not merely single excursions—may contribute to adverse renal and survival outcomes.

In contrast, a higher volatility index, reflecting intra-individual variability of trough concentrations, was inversely associated with AKI (discussed in more detail below). Our findings align with and extend current evidence from the literature [14,15,16,17]. Bellos et al. demonstrated a clear dose–toxicity relationship, reporting that trough levels ≥ 15 mg/L were linked to a significantly higher risk of AKI, with specificity increasing further at >20 mg/L [18]. Similarly, Gyamlani et al. observed that trough concentrations >20 mg/L nearly quadrupled the risk of AKI [19]. These reports support our observation that sustained vancomycin exposure above 15 mg/L, as captured by TIR, is harmful, even at lower thresholds than the traditionally cited >20 mg/L cut-off [20]. This emphasizes that cumulative exposure over time, rather than isolated trough values, drives nephrotoxicity and mortality. Notably, attributing AKI specifically to vancomycin remains challenging in critically ill patients due to multiple competing and time-varying renal insults, including sepsis, hypotension, and concomitant nephrotoxic therapies. While experimental and clinico-pathological data support a mechanistic basis of vancomycin nephrotoxicity—such as tubular injury, interstitial inflammation, and cast formation [21]—its independent contribution in clinical settings appears modest after accounting for confounding. In a marginal structural model adjusting for time-dependent factors, vancomycin exposure was associated with AKI primarily at higher concentrations, particularly above 20 mg/L [22]. Moreover, comparative studies suggest an approximately 2.5-fold increased AKI risk compared with alternative Gram-positive agents [23]. Together, these data support the interpretation that our findings reflect vancomycin-associated rather than exclusively vancomycin-induced AKI.

Our mortality findings resonate with the recent study by Chen et al., which showed that in critically ill older adults, higher trough concentrations were independently associated with both vancomycin-associated AKI and 30-day mortality, with the risk increasing markedly above ~19 mg/L [24]. Hou et al. reported that patients with mean VTCs of 15–20 mg/L and >20 mg/L had significantly higher ICU and hospital mortality compared with those with VTC < 10 mg/L [25]. Notably, maintaining VTCs of 15–20 mg/L throughout therapy did not improve survival [25]. These observations are consistent with our findings, in which sustained exposure above 15 mg/L (TIR _>15_) was associated with harm, while the lowest mortality occurred at mean trough levels of 8–12 mg/L. However, this mortality association must be interpreted cautiously given the retrospective single-center design, the high ICU mortality rate of 47.7%, and the absence of detailed illness severity indicators such as APACHE II or SOFA. In critically ill septic patients, higher vancomycin exposure may partly reflect reduced renal clearance, evolving organ dysfunction, or greater underlying disease severity rather than a direct toxic effect of vancomycin itself. Therefore, the observed association between TIR _>15_ and ICU mortality should be regarded as exploratory and hypothesis-generating, not as evidence of causality. Importantly, although residual confounding by disease severity cannot be fully excluded, emerging evidence suggests that the association between higher vancomycin exposure and adverse outcomes is not solely explained by impaired renal clearance. A large cohort study demonstrated that concentrations >25 mg/L were consistently associated with increased mortality and AKI, whereas lower ranges (15–20 mg/L) were associated with more favorable outcomes depending on disease severity [26]. In line with this, a meta-analysis including over 5000 patients reported that trough levels < 15 mg/L were associated with reduced all-cause mortality compared with higher concentrations [27].

At the same time, higher vancomycin exposure has been linked to improved microbiological response in selected settings, particularly in MRSA infections, underscoring a clinically relevant efficacy–toxicity trade-off [26,28]. Importantly, the proposed trough-derived metrics should be interpreted in relation to, and not as substitutes for, established pharmacokinetic/pharmacodynamic (PK/PD) targets such as AUC/MIC. The potential added value of TIR and the volatility index lies in their ability to describe longitudinal exposure patterns when repeated trough concentrations are already available, particularly in settings where formal Bayesian AUC estimation is not routinely implemented. Whereas AUC/MIC remains the guideline-recommended exposure target, TIR may help distinguish isolated supratherapeutic trough values from sustained or recurrent exposure above predefined thresholds, and the volatility index may capture intra-individual variability reflecting dynamic renal function or dose adaptation.

Taken together, these findings suggest a possible exposure range around 8–12 mg/L in this cohort. However, this “Goldilocks” window should be interpreted as an exploratory descriptive finding rather than a validated therapeutic target. The observed U-shaped pattern may reflect residual confounding, differences in illness severity, renal clearance, and infection-specific exposure requirements, particularly in severe infections where higher guideline-consistent targets may be needed. Thus, our findings support individualized exposure assessment rather than uniform trough-based thresholds.

The relationship between vancomycin exposure and renal function is further complicated by ARC, which we identified in 4.6% of our cohort, exclusively among younger patients. Previous studies have shown that ARC predisposes to subtherapeutic exposure and treatment failure [11,13], consistent with our finding that ARC was strongly associated with TIR _<10_. These data highlight the importance of renal function monitoring and individualized dosing strategies, particularly in younger, hyperdynamic patients. However, ARC was infrequent in our cohort, and estimates should be interpreted cautiously due to the small sample size.

As noted above, a novel but counterintuitive finding of our study is the inverse association between the volatility index and advanced AKI. This should not be interpreted as evidence that variability itself is protective. A more plausible explanation is that higher volatility reflected the absence of sustained supratherapeutic exposure, possibly due to earlier dose adjustments, interval modifications, or dynamic renal function changes during routine care. In contrast, persistently elevated trough concentrations may indicate prolonged accumulation and greater cumulative nephrotoxic exposure. This interpretation is consistent with pharmacokinetic evidence linking cumulative exposure (AUC) and prolonged high troughs to nephrotoxicity [15], whereas strategies that limit sustained elevation—such as AUC-guided or continuous infusion regimens—have shown lower AKI risk in meta-analyses [29,30,31,32]. Accordingly, the volatility index should be interpreted as an exploratory marker of non-sustained exposure and adaptive dosing dynamics rather than as a direct renal-protective factor. At present, the volatility index has limited bedside interpretability because no validated clinical thresholds exist, and its calculation requires serial trough measurements; therefore, it should be viewed as a retrospective descriptive marker rather than a stand-alone tool for real-time dose adjustment.

In our cohort, higher age was associated with a lower volatility index, a reduced probability of TIR _<10_, and a lower likelihood of ARC. These results are consistent with previous reports showing that ARC predominantly occurs in younger critically ill patients, especially those with trauma or sepsis [10], whereas older patients have reduced renal reserve and less dynamic clearance [33]. The lower volatility observed in older patients may reflect more stable renal function, but it could also indicate a diminished capacity to adapt to fluctuating drug exposure, thereby increasing susceptibility to sustained high concentrations.

We also observed that both current and historical detection of VRE were independently associated with advanced AKI. This observation aligns with studies demonstrating that VRE colonization or infection is a marker of poor outcomes and is associated with a higher risk of AKI in transplant and critically ill populations [34]. However, this association should not be interpreted as a causal link between vancomycin exposure and VRE occurrence. In our data, vancomycin exposure itself was not correlated with VRE carriage or infection rates. The observed relationship between VRE positivity and adverse outcomes most likely reflects higher illness severity, comorbidity burden, and cumulative antibiotic exposure rather than a direct drug effect. Accordingly, VRE status in this study should be viewed as a surrogate marker of clinical complexity rather than as a therapeutic endpoint or a variable influenced by vancomycin dosing. Although not previously emphasized in vancomycin exposure research, our findings suggest that microbiological risk factors such as VRE colonization or infection may serve as important modifiers of renal outcomes, primarily through their association with disease severity and prior antimicrobial exposure.

Taken together, our findings underscore the limitations of trough-based monitoring alone in critically ill patients and suggest that sustained vancomycin exposure above commonly used thresholds may be associated with adverse outcomes, influenced by patient-specific factors including age-related renal reserve and microbiological profiles. The trough-derived metrics applied, including TIR and the volatility index, should be considered exploratory descriptors of exposure dynamics rather than validated decision-making tools. While these measures may help to describe cumulative exposure patterns and intra-individual variability in retrospective analyses, this study does not provide evidence to support their direct implementation as stand-alone tools in routine clinical practice. In practical terms, their use would require serial TDM measurements and would therefore be most realistic in ICU patients undergoing prolonged vancomycin therapy with repeated trough monitoring. In such settings, TIR and volatility could potentially be calculated automatically from existing laboratory data within electronic medical records or antimicrobial stewardship programs. However, no prospectively validated actionable thresholds for TIR or the volatility index can currently be recommended. Accordingly, the observed association between TIR _>15_ and adverse outcomes should be interpreted as hypothesis-generating rather than as a clinically validated intervention threshold. Prospective studies comparing these trough-derived metrics with AUC-guided monitoring are required before they can be incorporated into dosing algorithms, particularly where AUC-guided monitoring is not readily available.

### Limitations

This study has several limitations. Its single-center, retrospective design inherently limits generalizability and precludes causal inference. ICU severity scores (e.g., SAPS, APACHE II) were not available, which limits adjustment for baseline illness severity and outcome interpretation. Moreover, ICU mortality is inherently multifactorial, and several clinically relevant confounders could not be comprehensively accounted for, including detailed severity-of-illness measures, comorbidity burden, infection severity, hemodynamic instability, organ support, nephrotoxic co-medication, and dynamic changes in renal function during therapy. Although age and sex were selected a priori for restricted multivariable adjustment to avoid overfitting given the limited number of events, this approach cannot exclude residual confounding. Therefore, the observed association between TIR _>15_ and ICU mortality should not be interpreted as causal, but rather as an exploratory association requiring confirmation in larger prospective cohorts with detailed severity adjustment. Observational studies are susceptible to selection bias, measurement error, and residual confounding, which cannot be fully mitigated by adjustment for key demographic factors such as age and sex. In addition, the relatively small sample size and limited number of outcome events reduced statistical power and precision. This is reflected by the wide confidence intervals around several odds ratio estimates, indicating uncertainty regarding the exact magnitude of the observed associations. Therefore, although the direction and consistency of the findings are clinically relevant and supported by bootstrap sensitivity analyses, the effect estimates should be interpreted cautiously and considered exploratory rather than confirmatory. Critically ill patients present multiple time-varying risk factors for AKI and mortality that could not be fully captured in this retrospective analysis, including cumulative exposure to concomitant nephrotoxic drugs such as aminoglycosides, loop diuretics, vasopressors, nonsteroidal anti-inflammatory drugs, contrast agents, and other potentially nephrotoxic antimicrobial combinations. Baseline comorbidities such as diabetes and hypertension may further modify vancomycin nephrotoxicity in ways not fully captured by our analysis.

Causal attribution of AKI to vancomycin is inherently limited due to overlapping injury from sepsis, hypotension, and polypharmacy. Associations between TIR _>15_ and adverse outcomes may partially reflect reverse causality, as patients with impaired renal clearance accumulate higher vancomycin concentrations and underlying disease severity may independently contribute to mortality. Trough-based monitoring is an imperfect surrogate for total exposure, and our TIR and volatility metrics are novel, unvalidated descriptors of exposure dynamics that require prospective validation before clinical implementation.

Methodological limitations include incomplete reconstruction of detailed vancomycin dosing histories, including loading doses, maintenance doses, exact dosing intervals, interval modifications, and dose adjustments over time, as well as lack of peak concentration data and inability to confirm steady-state conditions for each trough measurement. In addition, heterogeneous trough sampling intervals limited temporal inference and precluded reliable reconstruction of time-weighted exposure or AUC. The exclusive use of intermittent infusion further limits extrapolation to continuous infusion or AUC-guided dosing strategies. Continuous infusion and AUC-guided strategies have been associated with lower AKI risk in meta-analyses; extrapolation from intermittent dosing is therefore limited, highlighting the need for prospective evaluation. ARC was infrequent (4.6%), and estimates in this subgroup should be interpreted cautiously due to the small sample size. Microbiological outcome data were limited, preventing assessment of efficacy-toxicity trade-offs. Patients with more frequent trough monitoring may differ systematically from those monitored less intensively, introducing potential selection bias.

Finally, the cohort was restricted to critically ill ICU patients, and post-ICU or long-term outcomes were unavailable, limiting conclusions to short-term in-ICU effects. The study period spans a decade ago (2010–2015); although selected for completeness of dosing records, this may limit generalizability to contemporary practice. Nonetheless, these findings provide robust, hypothesis-generating insights into trough-derived exposure dynamics in critically ill septic patients, and highlight areas for future prospective evaluation.

## 4. Materials and Methods

### 4.1. Patient Selection and Study Design

The retrospective analysis was conducted at a tertiary care center (University Hospital Essen, Essen, Germany). Medical records of 109 critically ill patients who received vancomycin therapy for septic infections in the ICU between 2014 and 2016 were reviewed. Patients were eligible for inclusion if they were at least 18 years of age, admitted to the ICU with a diagnosis of sepsis or septic shock according to international consensus definitions, treated with intravenous vancomycin for a minimum of 48 h, and had complete TDM data with at least two documented vancomycin serum concentrations available for exposure assessment. Patients with incomplete records, prior renal replacement therapy before initiation of vancomycin, or missing TDM data were excluded from the analysis.

All patients fulfilled the Sepsis-3 criteria for sepsis or septic shock, defined by life-threatening organ dysfunction with an increase in the Sequential Organ Failure Assessment (SOFA) score of ≥2 points; septic shock required vasopressor support to maintain a mean arterial pressure ≥ 65 mmHg and serum lactate > 2 mmol/L despite adequate fluid resuscitation [35]. In addition, sepsis diagnosis was confirmed retrospectively by review of the medical records, including documented clinical suspicion or diagnosis of infection, microbiological and/or radiological evidence where available, identified infection focus, initiation of antimicrobial therapy, and corresponding organ dysfunction during the index ICU stay.

The primary outcomes were all-cause ICU mortality and AKI during the index ICU stay. Mortality was not adjudicated as infection-related mortality because causes of death in critically ill septic patients are often multifactorial and could not be reliably classified retrospectively.

AKI was defined and staged according to the Kidney Disease: Improving Global Outcomes (KDIGO) guidelines based on serum creatinine changes: stage I (1.5–1.9-fold increase), stage II (2.0–2.9-fold increase), and stage III (≥3-fold increase or initiation of renal replacement therapy) [36]. Urine output criteria were not consistently available in the retrospective dataset and were therefore not used for AKI staging. Baseline creatinine was defined as the last available value prior to ICU admission or, if unavailable, the admission value. In this study, renal function was estimated using the 2021 Chronic Kidney Disease Epidemiology (CKD-EPI) Collaboration equation [37].

Vancomycin was administered as intermittent intravenous dosing according to institutional standard practice, with dosing intervals adjusted based on renal function, measured trough concentrations, and clinical judgment. Only documented pre-dose trough concentrations obtained during ongoing vancomycin therapy were included; however, exact sampling times varied in routine ICU care and could not be fully standardized or reconstructed retrospectively. Because of the retrospective design, steady-state conditions could not be reliably confirmed for each individual trough measurement, and dosing regimens were not assumed to remain stable throughout therapy. Dose adjustments and interval modifications were performed as part of routine clinical care. Dynamic changes in renal function during therapy were assessed through serial serum creatinine and eGFR measurements; however, due to incomplete reconstruction of exact dosing times, interval changes, and dose adjustments, these dynamics could not be incorporated into formal time-updated pharmacokinetic or dose-adjustment models. Detailed vancomycin dosing histories—including loading doses, maintenance doses, dosing intervals, dose adjustments, and precise sampling timestamps—were not consistently available and could therefore not be systematically analyzed. A total of 720 trough concentrations were analyzed (median 5 per patient, interquartile range 3–9), reflecting real-world exposure before and during AKI development.

Serum or plasma VTCs were measured as part of routine TDM in the central laboratory of the University Hospital Essen using the QMS^®^ Vancomycin assay on an automated clinical chemistry analyzer, according to the manufacturer’s instructions (Thermo Fisher Scientific/B·R·A·H·M·S GmbH, Hennigsdorf, Germany). The assay is a homogeneous particle-enhanced turbidimetric immunoassay based on competitive inhibition between vancomycin in the patient sample and vancomycin-coated microparticles for anti-vancomycin antibody binding sites. Microparticle agglutination is inversely related to the vancomycin concentration and was quantified photometrically. Calibration was performed using six QMS^®^ Vancomycin calibrators covering 0–100 µg/mL. The analytical measuring range was 2.5–100 µg/mL, with a lower limit of quantitation of 2.0 µg/mL. Quality control was performed according to the manufacturer’s recommendations and local laboratory procedures. Results were reported in µg/mL, equivalent to mg/L.

We calculated vancomycin exposure using VTCs to derive the TIR, as well as a volatility index reflecting intra-individual variability. AUC/MIC-guided exposure targets were considered as the current guideline-recommended standard; however, AUC/MIC could not be reliably reconstructed retrospectively and was therefore not included as an exposure variable. Accordingly, trough-derived metrics were evaluated as pragmatic exploratory surrogates rather than replacements for AUC-guided monitoring. In line with current consensus guidelines and previous pharmacokinetic studies, clinically relevant ranges were defined as 10–20 mg/L, 15–20 mg/L, and 15–25 mg/L, whereas concentrations <10 mg/L, >20 mg/L, or >25 mg/L were considered subtherapeutic or supratherapeutic, respectively [2,14,17,38]. In this study, TIR was defined as the proportion of available trough measurements within these predefined ranges, reflecting the relative frequency of concentrations within or above therapeutic targets. Accordingly, it represents a trough-sampling-based, proportion-derived exposure metric rather than a true time-dependent or time-weighted measure. Because sampling frequency may influence this metric, TIR values were interpreted as pragmatic descriptors of observed trough exposure patterns and not as direct estimates of continuous time spent within a concentration range. This proportion-based approach was chosen due to the retrospective design and heterogeneous sampling intervals, which precluded reliable reconstruction of continuous time-above-threshold or AUC data. Despite this limitation, the primary TIR exposure metric was defined as TIR _>15_, reflecting the proportion of trough concentrations exceeding 15 mg/L. This threshold was selected a priori based on prior studies demonstrating an increased risk of nephrotoxicity at trough levels ≥ 15 mg/L, without consistent evidence of improved clinical outcomes [14,17,39,40]. Additional TIRs (10–20, 15–20, 15–25, <10, >20, and >25 mg/L) were evaluated for exploratory and descriptive purposes only.

### 4.2. Statistical Methods

All data were extracted from the hospital information system, preprocessed in Microsoft Excel, and anonymized. Statistical analyses were performed using GraphPad Prism (version 10.6; GraphPad Software, San Diego, CA, USA) and Python (version 3.11; Python Software, Wilmington, DE, USA). Continuous variables with a normal distribution are presented as mean ± standard deviation (SD), whereas non-normally distributed variables are expressed as median and interquartile range (Q1–Q3). Categorical variables are summarized as absolute numbers (n) and percentages (%). Comparisons between groups were conducted using one-way ANOVA or Kruskal–Wallis test, as appropriate. Categorical data were compared using the chi-square test. Analyses followed a predefined stepwise approach. Descriptive statistics summarized baseline characteristics, microbiological findings, renal function, and vancomycin exposure metrics; age-tertile analyses were conducted for exploratory purposes only. Associations between exposure parameters (mean trough concentration, TIR _>15_, volatility index) and outcomes (ICU mortality and AKI) were primarily assessed using univariable logistic regression and reported as the main results. Given the limited number of events, multivariable models were applied in a restricted, exploratory manner with adjustment for age and sex selected a priori to avoid overfitting. Linear regression was used for continuous outcomes, and additional patient-related and microbiological variables (e.g., renal function, ARC, and culture findings) were evaluated in exploratory analyses. Model robustness was assessed using nonparametric bootstrap resampling to evaluate the stability of regression estimates in the context of the limited sample size and event numbers. A total of 1000 bootstrap iterations was selected as a pragmatic number commonly used to obtain stable confidence interval estimates while maintaining computational feasibility. Restricted cubic spline models were applied to explore potential non-linear exposure–outcome relationships. A two-sided *p*-value < 0.05 was considered statistically significant.

## 5. Conclusions and Future Research Directions

In critically ill septic patients receiving vancomycin, sustained trough concentrations > 15 mg/L (TIR _>15_) were associated with increased ICU mortality and severe AKI (stage II–III), whereas the most favorable outcomes were descriptively observed at mean trough levels of approximately 8–12 mg/L. These findings are consistent with prior evidence linking higher vancomycin exposure to nephrotoxicity and mortality; however, the observed 8–12 mg/L range remains exploratory and may not apply to severe infections, such as VAP or sCAP/HAP, where higher guideline-consistent exposure targets may be required.

Given the exploratory nature of TIR and volatility metrics, these observations should be interpreted cautiously, and do not replace guideline-recommended AUC-guided monitoring. Volatility was inversely associated with AKI, likely reflecting adaptive dosing dynamics rather than a direct protective effect.

Future research should focus on prospective, multicenter validation of TIR and volatility metrics alongside AUC-guided dosing; investigation of disease severity-stratified vancomycin targets; incorporation of novel biomarkers (e.g., urinary Kidney Injury Molecule-1 (KIM-1), serum cystatin C [41]) for earlier AKI detection; pharmacokinetic modeling to identify patient-specific predictors of sub- or supratherapeutic exposure; and prospective evaluation of the proposed “Goldilocks window” of 8–12 mg/L. These studies will be essential to refine individualized dosing strategies and determine whether trough-derived patterns can provide clinically useful guidance when AUC monitoring is not available.

## Figures and Tables

**Table 1:** Baseline characteristics and vancomycin exposure parameters across age tertiles.

Variable	Total (n = 109)	Younger Tertile: ≤54 Years (n = 37)	Middle Tertile: 55–67 Years (n = 37)	Older Tertile: ≥68 Years (n = 35)	*p*-Value
Age, years	59.8 ± 13.5	45.1 ± 9.1	61.4 ± 3.9	74.2 ± 5.5	—
Male sex	76 (69.7)	29 (78.4)	21 (56.8)	26 (74.3)	0.1000
ICU mortality	52 (47.7)	18 (48.6)	19 (51.4)	15 (42.9)	0.7633
AKI stage I	11 (10.1)	1 (2.7)	5 (13.5)	5 (14.3)	0.1843
AKI stage II–III	48 (44.0)	15 (40.5)	14 (37.8)	19 (54.3)	0.3243
Detection of Gram-negative bacteria	22 (20.2)	6 (16.2)	9 (24.3)	8 (22.9)	0.6616
Detection of vancomycin-susceptible bacteria	59 (54.1)	23 (62.2)	23 (62.2)	13 (37.1)	**0.0493**
Detection of VRE currently	7 (6.4)	3 (8.1)	1 (2.7)	3 (8.6)	0.5231
Detection of VRE ever	14 (12.8)	4 (10.8)	6 (16.2)	4 (11.4)	0.7501
Treatment duration	6 (3–13)	6 (4–11)	7 (4–16)	7 (2–11)	0.4188
Mean serum creatinine, mg/dL	2.2 ± 1.7	2.3 ± 2.2	2.1 ± 1.4	2.4 ± 1.7	0.6825
Mean eGFR, mL/min/1.73 m^2^	53.2 ± 38.1	69.6 ± 45.7	49.4 ± 31.1	39.9 ± 30	**0.0027**
Mean vancomycin trough, mg/L	13.1 ± 4.7	12.3 ± 4.7	13.1 ± 4.1	13.8 ± 5.2	0.4002
TIR _10–20_	0.51 ± 0.35	0.45 ± 0.37	0.55 ± 0.34	0.53 ± 0.34	0.4224
TIR _>20_	0.12 ± 0.2	0.09 ± 0.15	0.13 ± 0.19	0.15 ± 0.25	0.3415
TIR _<10_	0.37 ± 0.35	0.47 ± 0.37	0.32 ± 0.33	0.32 ± 0.33	0.1100
TIR _15–20_	0.22 ± 0.26	0.18 ± 0.25	0.25 ± 0.27	0.23 ± 0.28	0.5260
TIR _<15_	0.66 ± 0.32	0.73 ± 0.29	0.63 ± 0.32	0.62 ± 0.35	0.2883
TIR _15–25_	0.30 ± 0.31	0.23 ± 0.28	0.34 ± 0.30	0.33 ± 0.34	0.2314
TIR _>25_	0.04 ± 0.11	0.04 ± 0.11	0.03 ± 0.09	0.05 ± 0.12	0.8254
TIR _>15_	0.34 ± 0.32	0.27 ± 0.29	0.37 ± 0.32	0.38 ± 0.35	0.2883
TIR _10–15_	0.31 ± 0.28	0.30 ± 0.29	0.32 ± 0.26	0.32 ± 0.29	0.9188
TIR outside 10–15	0.69 ± 0.28	0.70 ± 0.29	0.68 ± 0.26	0.68 ± 0.29	0.9188
Volatility index	0.31 ± 0.27	0.37 ± 0.29	0.31 ± 0.28	0.26 ± 0.21	0.2569
ARC	5 (4.6)	5 (4.6)	0 (0.0)	0 (0.0)	**0.0061**

Data are presented as mean ± standard deviation (SD), median (Q1–Q3), or n (%), as appropriate. *p*-values are derived from one-way ANOVA for normally distributed continuous variables, Kruskal–Wallis test for non-normally distributed variables, and Chi-square test for categorical variables. Age-stratified comparisons are presented for descriptive and exploratory purposes only. TIR is reported as a dimensionless proportion (range 0–1) of trough measurements within the specified concentration ranges. Significant differences are shown in bold. ARC = augmented renal clearance; AKI = acute kidney injury; eGFR = estimated glomerular filtration rate; TIR = time in therapeutic range; VRE = Vancomycin-Resistant *Enterococcus*.

## Data Availability

Data supporting the findings of this study can be obtained from the corresponding author upon reasonable request and in accordance with institutional and ethical regulations.

## Supplementary Materials

The following supporting information can be downloaded at: https://www.mdpi.com/article/10.3390/antibiotics15060573/s1, Table S1. Sources of vancomycin-susceptible isolates. Table S2. Sources of Gram-negative isolates. Table S3. Logistic regression analysis showing no significant associations with ICU mortality.

## Abbreviations

AKI	Acute Kidney Injury
APACHE II	Acute Physiology and Chronic Health Evaluation II
ARC	Augmented Renal Clearance
AUC	Area Under the Concentration–Time Curve
CI	Confidence Interval
CKD-EPI	Chronic Kidney Disease Epidemiology Collaboration
eGFR	Estimated Glomerular Filtration Rate
HAP	Hospital-Acquired Pneumonia
ICU	Intensive Care Unit
KDIGO	Kidney Disease: Improving Global Outcomes
MIC	Minimum Inhibitory Concentration
MRSA	Methicillin-Resistant *Staphylococcus aureus*
OR	Odds Ratio
PK/PD	Pharmacokinetic/Pharmacodynamic
SAPS	Simplified Acute Physiology Score
sCAP	severe Community-Acquired Pneumonia
SD	Standard Deviation
SOFA	Sequential Organ Failure Assessment
TDM	Therapeutic Drug Monitoring
TIR	Time in Therapeutic Range
VAP	Ventilator-Associated Pneumonia
VRE	Vancomycin-Resistant *Enterococcus*
VTC	Vancomycin Trough Concentration
