# Supplementary material for: Vancomycin Exposure Dynamics and Clinical Outcomes in Critically Ill Patients: A Retrospective Cohort Study
Source: Antibiotics (Basel). 2026 Jun 4;15(6):573. doi: 10.3390/antibiotics15060573 (PMC13296325; doi:10.3390/antibiotics15060573)
Supplement: Supplementary file 1 [file antibiotics-15-00573-s001.zip › antibiotics-4315380-supplementary.pdf]

Supplementary Table S1.

Vancomycin-susceptible isolates	Isolate source							
	**BC**	**Ascites**	**Surgical wound**	**Sputu m**	**Pleura**	**Gallbladde r**	**Urine**	**Total**
Staphylococcus epidermidis	37	6		1				44
Enterococcus faecium	21	7	3		1	2	2	36
Staphylococcus haemolyticus	1							1
Lactobacillus paracasei	1							1
Enterococcus faecalis	14	1						15
Staphylococcus aureus (MSSA)	1		1					2
Staphylococcus aureus (MRSA)	2	1	1					4
Streptococcus agalactiae	1							1

Supplementary Table S1. Sources of vancomycin-susceptible isolates.
The table summarizes the distribution of vancomycin-susceptible bacteria isolated from various clinical specimens. Numbers represent the frequency of isolates obtained from blood cultures (BC), ascitic fluid, surgical wound swabs, sputum, pleural effusion, gallbladder fluid, and urine. Only isolates tested as susceptible to vancomycin are included. MSSA = Methicillin-susceptible *Staphylococcus aureus*, MRSA = Methicillin-resistant *Staphylococcus aureus*.

Supplementary Table S2.

Gram-negative isolates	Isolate source				
	**BC**	**Ascites**	**Urine**	**BAL**	**Total**
Enterobacter cloacae	2				2
Escherichia coli	7	1	2	1	11
Klebsiella oxytoca	2				2
Escherichia coli (3MRGN)	3				3
Serratia liquefaciens	1				1
Enterobacter aerogenes	2				2
Enterobacter aerogenes (3MRGN)	1				1
Klebsiella pneumoniae (3MRGN)	2				2
Acinetobacter radioresistens	1				1
Klebsiella pneumoniae	2		1		3
Pseudomonas aeruginosa	2		1		3

Supplementary Table S2. Sources of Gram-negative isolates.
The table presents gram-negative bacteria isolated from various clinical specimens. Numbers represent the frequency of isolates obtained from blood cultures (BC), ascitic fluid, urine, and bronchoalveolar lavage (BAL). Multi-resistant bacteria (3MRGN) are indicated accordingly.

Supplementary Table S3.

**Variable**	**OR**	**95% CI (Lower–Upper)**	**p-value**
Age	1.009	0.9808–1.038	0.5410
Sex (Female)	1.768	0.7784–4.098	0.1737
Treatment duration (days)	0.9817	0.9334–1.029	0.4434
Volatility index	0.9071	0.2096–3.833	0.8936
AKI stage I	0.5952	0.1481–2.101	0.4237
AKI stage II–III	1.591	0.7451–3.433	0.2306
ARC	1.684	0.2686–13.18	0.5727
VRE detection (current)	0.416	0.05775–2.027	0.2861
Any history of VRE	1.220	0.3879–3.837	0.7299
Detection of Gram-negative bacteria	0.4848	0.1783–1.239	0.1318

Supplementary Table S3. Logistic regression analysis showing no significant associations with ICU mortality.
The table summarizes univariate logistic regression models evaluating potential predictors of ICU mortality, including demographic variables, treatment-related parameters, renal function (AKI stages, ARC), and microbiological findings (Gram-negative bacteria, VRE status). None of the tested variables showed a statistically significant association with ICU mortality. Results are presented as odds ratios (OR) with 95% confidence intervals (CI). AKI = acute kidney injury; ARC = augmented renal clearance; VRE = vancomycin-resistant *Enterococcus*.
